# Whole-body MRI for the investigation of joint involvement in inflammatory arthritis

**DOI:** 10.1007/s00256-023-04515-0

**Published:** 2023-11-22

**Authors:** Jane Freeston, Matthew Marzetti, Neal Larkman, Emma Rowbotham, Paul Emery, Andrew Grainger

**Affiliations:** 1 Leeds NIHR Musculoskeletal Biomedical Research Centre, Leeds, UK; 2 Leeds Institute of Rheumatic and Musculoskeletal Medicine, University of Leeds, Leeds, UK; 3 Department of Medical Physics, Leeds Teaching Hospitals NHS Trust, Leeds, UK; 4 Department of Radiology, Harrogate and District NHS Foundation Trust, Harrogate, UK; 5 Department of Radiology, Leeds Teaching Hospitals NHS Trust, Leeds, UK; 6 Department of Radiology, Cambridge University Hospitals NHS Foundation Trust, Cambridge, UK

**Keywords:** Whole-body MR imaging, Magnetic resonance imaging, Inflammatory arthritis

## Abstract

**Objectives:**

This study aimed to develop a novel whole-body MRI protocol capable of assessing inflammatory arthritis at an early stage in multiple joints in one examination.

**Materials and methods:**

Forty-six patients with inflammatory joint symptoms and 9 healthy volunteers underwent whole-body MR imaging on a 3.0 T MRI scanner in this prospective study. Image quality and pathology in each joint, bursae, entheses and tendons were scored by two of three radiologists and compared to clinical joint scores. Participants were divided into three groups based on diagnosis at 1-year follow-up (healthy volunteers, rheumatoid arthritis and all other types of arthritis). Radiology scores were compared between the three groups using a Kruskal-Wallis test. The clinical utility of radiology scoring was compared to clinical scoring using ROC analysis.

**Results:**

A protocol capable of whole-body MR imaging of the joints with an image acquisition time under 20 min was developed with excellent image quality. Synovitis scores were significantly higher in patients who were diagnosed with rheumatoid arthritis at 12 months (*p* < 0.05). Radiology scoring of bursitis showed statistically significant differences between each of the three groups—healthy control, rheumatoid arthritis and non-rheumatoid arthritis (*p* < 0.05). There was no statistically significant difference in ROC analysis between MRI and clinical scores.

**Conclusion:**

This study has developed a whole-body MRI joint imaging protocol that is clinically feasible and shows good differentiation of joint pathology between healthy controls, patients with rheumatoid arthritis and patients with other forms of arthritis.

## Introduction

Imaging is a crucial step in the pathway for investigating and monitoring patients with joint disease. Both magnetic resonance imaging (MRI) and ultrasound have a place in this pathway, and the imaging findings are well described [1]. The importance of imaging is in early diagnosis of inflammatory arthropathy allowing early instigation of treatment.

Even with the most sensitive imaging techniques, making a reliable clinical diagnosis early in the disease pathway presents challenges due to the subtle nature of early inflammatory changes. Differentiating this from non-inflammatory disease, as well as the timely classification of the type of inflammatory arthritis (AI), represents a significant challenge.

Ultrasound is often used in clinical practice as a screening tool in patients with early disease, allowing multiple joints to be scanned at the same sitting. MRI is usually reserved for individual symptomatic joints or as an assessment option in the research setting. Whilst MRI is proven to be an accurate diagnostic tool and improves upon the sensitivity of clinical examination [2, 3], its routine usage has been limited by logistic difficulties of scanning multiple joints at one appointment. Consequently, until recently, scanning of multiple joints using whole-body MRI (WBMRI) has largely been reserved for the research setting.

Knowing the overall extent of joint involvement is recognised as an important part of the diagnosis and management of rheumatological conditions [4]. In clinical trials of rheumatoid arthritis with imaging endpoints, traditionally a unilateral wrist and metacarpophalangeal joints 2–5 have been imaged based on feasibility and maximum diagnostic yield [5, 6]. This approach does not allow for detecting findings in other asymptomatic joints, or establishing patterns of joint involvement. WBMRI allows multiple joint assessment from one imaging study, and its use in axial spondyloarthropathies, including the assessment of early disease [7] and response to treatment [8], has evolved to include detection and assessment of response [9–11].

Traditionally, WBMRI covers the entire chest, abdomen and pelvis as well as the joints. We have developed a novel whole-body joint-based imaging protocol, optimising imaging of the peripheral joints and the axial skeleton, whilst also minimising scan time and maximising patient comfort.

We propose that this whole-body joint-based MRI protocol will allow imaging of multiple joints in one appointment, is acceptable to patients and will allow increased accuracy in detecting and categorising early inflammatory disease. Both patients and healthy controls were included to test the feasibility of the protocol in a clinical setting and whether imaging findings correlate with clinical examination, as well as to allow a comparison of the performance at MRI to clinical findings.

## Methods

This prospective study recruited 46 consecutive patients presenting for the first time to the Early Arthritis Clinic at Leeds Teaching Hospitals NHS Trust with inflammatory joint symptoms between June 2010 and October 2016 and 10 healthy controls (HC), although only 9 were included in the study. Participants were recruited into the IA disease continuum (IACON) study, which was approved by the local research ethics committee, and all participants provided written informed consent.

All patients had 1 h or more of early morning stiffness and no clinician-confirmed diagnosis at presentation. WBMRI and clinical assessment (tender and swollen joint assessment) were performed at baseline, with a final clinical diagnosis of IA or not recorded at 12 months. The final clinical diagnosis was made by clinicians that were not involved in the study and was made as part of the patient’s clinical care outside of the research study. The results of the baseline MRIs were not available to the clinician making the clinical diagnosis at 1-year follow-up. MRIs were evaluated solely for research purposes. Patients with evidence of IA were classified as either rheumatoid arthritis (RA) or non-RA. A scoring atlas was devised by the radiologists to ensure reproducibility of the scores which was used by all radiologists.

### MR imaging

**Fig. 1:**
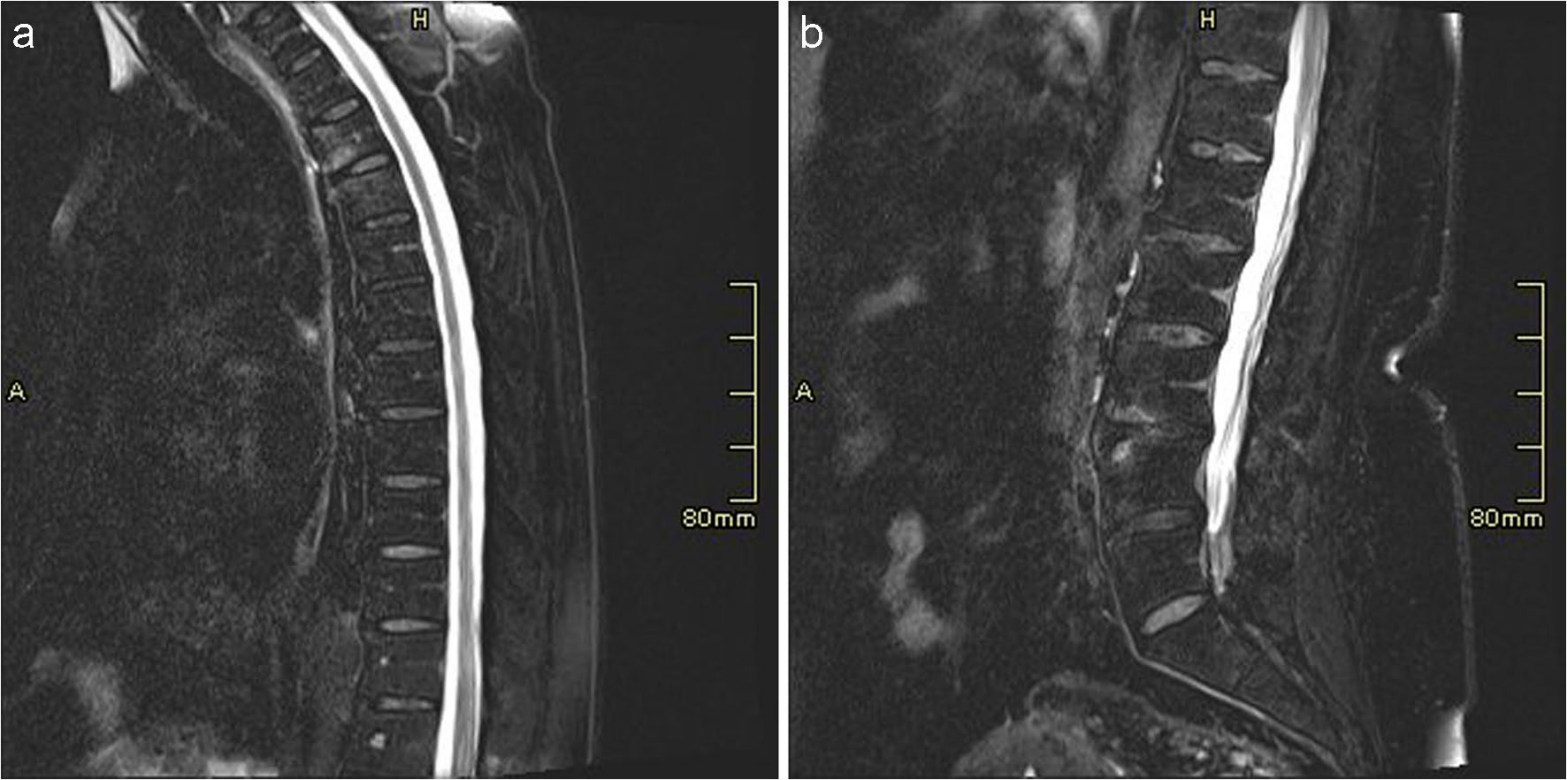
T2-FS sagittal imaging of the (**a**) thoracic and (**b**) lumbar spines in a normal control patient. These water-sensitive sequences allow imaging of the entire T and L spines which would show early signs of inflammatory arthropathy

**Fig. 2:**
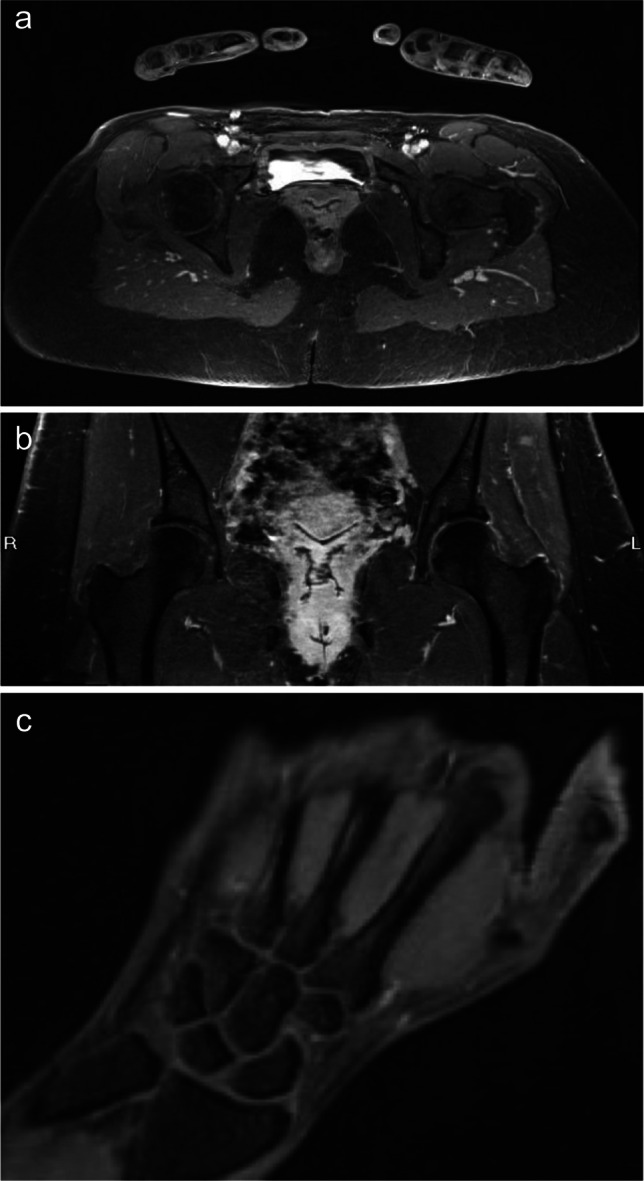
**a** Water-sensitive 3D VIBE Dixon imaging of the hips and hands which were imaged simultaneously in the same field of view (FOV) using two 4-channel small flex coils and the spine coil. The hand images are then reconstructed for reading in 3 orthogonal planes. **b** Reconstruction of the water-sensitive 3D VIBE Dixon axial imaging through the pelvis into a coronal plane showing both hip joints in a control patient. **c** Reconstruction of the water-sensitive 3D VIBE Dixon imaging to show a coronal image of the wrist and hand. No synovitis or erosive change is seen in this case

All MRI examinations were performed on a 3.0 Tesla MRI system (Magnetom Verio, Siemens Healthineers AG, Erlangen, Germany). Imaging of the spine, sacroiliac joints (SIJs), shoulders, hips, hands, knees and feet was undertaken with specific body area coils selected for each joint. The cervical spine, thoracic spine, lumbar spine and SIJ joints were imaged individually using a 16-channel spine coil (Fig. 1). After completion of spine imaging, both shoulders were imaged simultaneously using an 8-channel body coil and the superior elements of a 16-channel spine coil. The hips and hands were imaged simultaneously in the same field of view (FOV) using two 4-channel small flex coils and the spine coil (Fig. 2). The hands were positioned in a pronated position resting on the lower pelvis. Both knees were imaged with a 16-channel peripheral angiography (PA) coil, and finally, the ankles and feet were imaged in the same PA coil.

### MRI protocol

**Fig. 3:**
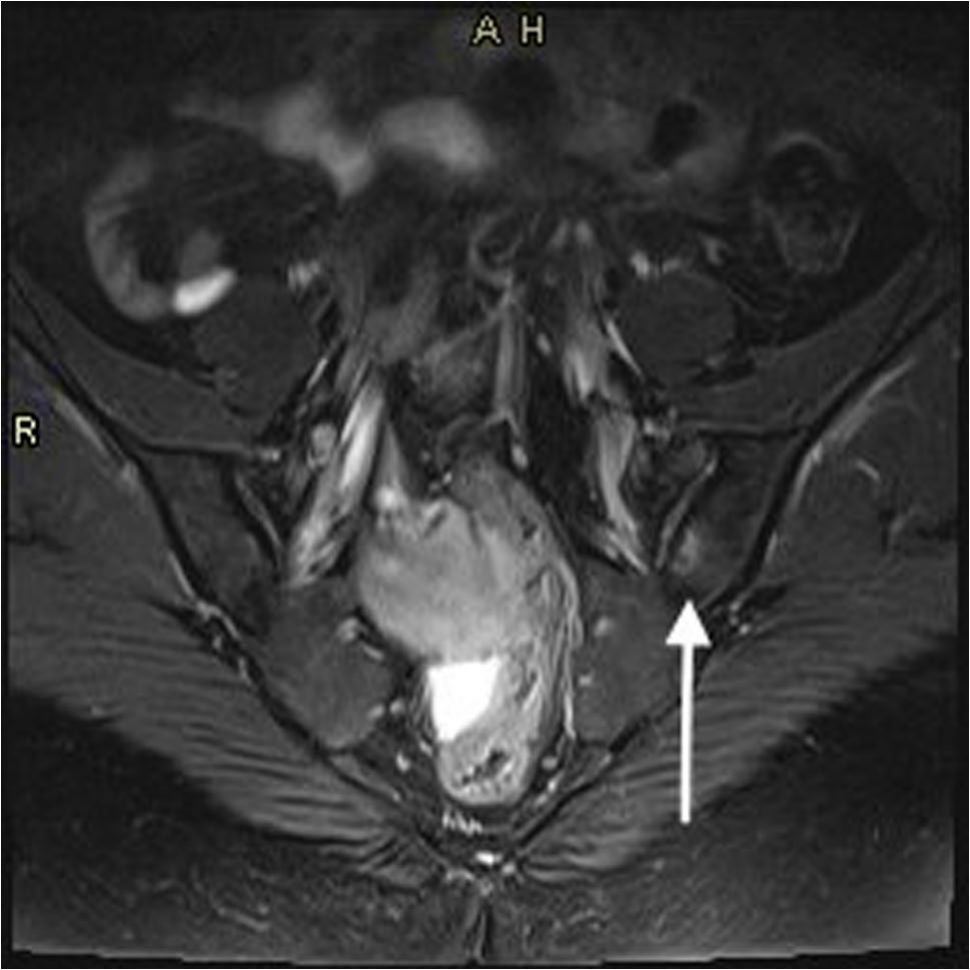
SI joint imaging in a patient with undifferentiated inflammatory arthritis. The figure shows oblique T2-W FS FSE image of the sacroiliac joints showing early left-sided subchondral bone marrow oedema (white arrow)

**Fig. 4:**
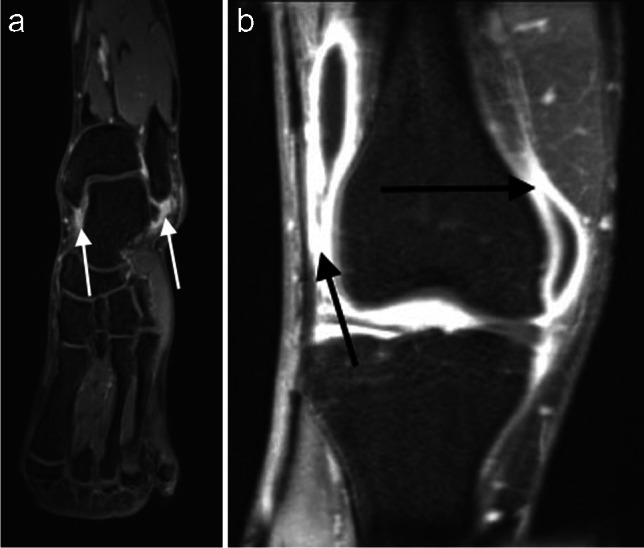
**a** Water-sensitive T1-weighted VIBE Dixon post-gadolinium image of the ankle in a patient with rheumatoid arthritis with synovitis of the tibio talar joint (white arrows). **b** Coronal water-sensitive VIBE Dixon post-gadolinium image of the knee in the same patient with marked synovitis seen within the joint (black arrows) and a moderate sized joint effusion

**Fig. 5:**
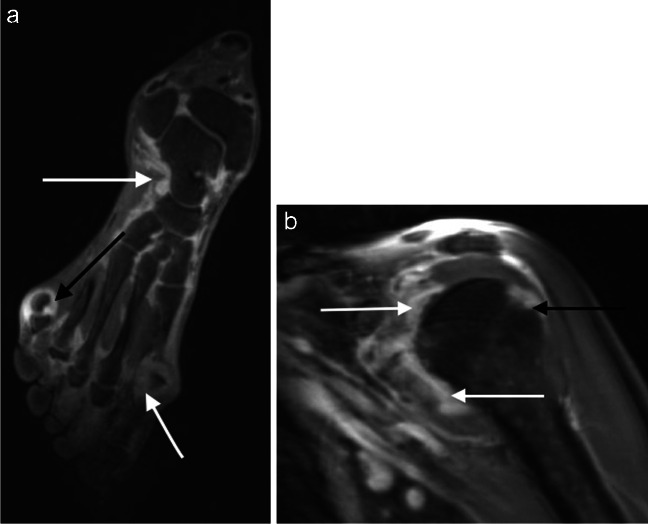
**a** Coronal water-sensitive post-gadolinium imaging of the foot in a patient with rheumatoid arthritis showing synovitis (white arrows) and also erosion (black arrows). **b** Coronal water-sensitive post-gadolinium imaging of the shoulder in the same patient with further extensive synovitis throughout the glenohumeral joint (white arrow) and also erosion of the humeral head (black arrow)

A sagittal T2-weighted (T2-W) fat-saturated (FS) fast spin echo (FSE) imaging sequence was used to image the spine. An oblique T2-W FS FSE sequence was used when imaging the SIJs (Fig. 3). All other joints were imaged after administration of gadolinium-based contrast using an axial T1-weighted (T1-W) 3D Dixon Volume Interpolated Breath-hold Examination (VIBE) spoiled gradient sequence, providing in-phase, out-of-phase, fat-only and water-only images. The 3D sequence was acquired with near isotropic voxel size and allowed multiplanar reconstructions (MPR) of each area for reading (Fig. 2). Scan parameters are shown in Table 1, with a total image acquisition time of 17 min and 5 s. Typical images acquired for each joint are shown in Figs. 1, 2, 3, 4 and 5.

**Table 1:** MR imaging parameters

Parameter	Spine	SIJ	Shoulders	Hips and hands	Knees	Feet
Sequence type	2D FSE FS	2D FSE FS	3D Vibe Dixon	3D Vibe Dixon	3D Vibe Dixon	3D Vibe Dixon
TR/TE (ms)	3100/105	4580/107	11/2.5 and 3.7	6.4/2.5 and 3.7	10/2.5 and 3.7	6.4/2.5 and 3.7
Flip angle (°)	90/160	90/160	15	15	20	15
Resolution (mm)	1.2 × 0.9	1.1 × 0.9	1.0 × 1.0	0.8 × 0.8	0.8 × 0.8	0.8 × 0.8
Field of view (mm)	285 × 285	220 × 220	281 × 500	303 × 404	202 × 404	384 × 424
Slice thickness (gap) (mm)	3 (0.3)	3 (0.9)	0.6 (0)	0.6 (0)	0.5 (0)	0.5 (0)
Bandwidth (Hz/px)	260	260	488	488	488	488
Parallel imaging factor	none	none	2	2	2	2
Acquisition time	1 min 8 s	1 min 22 s	3 min 1 s	3 min 3 s	2 min 58 s	3 min 17 s

Spine imaging sequence was repeated three times to image cervical, thoracic and lumbar spine

### MRI scoring

MRI scoring was performed by three experienced radiologists for 10 joint areas (AG, RH and ER with 14, 19 and 8 years’ experience, respectively). Two of the three scored each case—with the most experienced reader reading every case. At the time of scoring, the readers had the ability to perform 3D MPR reconstructions of the 3D datasets and had access to all the sequences. Water-sensitive images were used for assessment of inflammatory changes and the fat-sensitive and in-phase images for assessment of osteitis. Out-of-phase images were not reviewed as part of this assessment. Scoring was undertaken independently by each scorer who was blinded to whether the subject was a healthy control or patient and to the disease classification of the patients. In cases of discrepancy between reader scores, the final score given was determined by consensus.

1. Appendicular

- Joints (synovitis and erosions): using the post-gadolinium VIBE-Dixon images, the sternoclavicular, glenohumeral, wrist, metacarpophalangeal (MCP), proximal interphalangeal, hip, knee, ankle, mid/hind foot and metatarsophalangeal (MTP) joint areas were scored. The presence of synovitis and erosion was recorded (Figs. 4 and 5). The thumb carpometacarpal (CMC), 1st MCP and 1st hallux MTP joints were excluded due to the high prevalence of osteoarthritis in this joint in the general population, which may lead to false positive results for synovitis and erosion. Knee synovitis of less than 2 mm diameter was also excluded (due to the high likelihood of degenerative change as the underlying aetiology at these joints). The interphalangeal joints of the hands and feet were evaluated. Each joint was scored in a binary fashion with 0 indicating no pathology and 1 indicating a positive finding. A summed score of 0–22 was obtained for synovitis and erosion separately.

b) Enthesitis: the presence or absence of bone oedema at enthesis sites was scored bilaterally, using the post-gadolinium water-sensitive VIBE sequences, at the 1st costochondral junction, the anterior superior iliac spine, anterior inferior iliac spine, the ischial tuberosity, the femoral insertion of the medial collateral ligament, the femoral and tibial attachments of the lateral collateral ligament attachment site, the quadriceps and patellar tendon insertions on the patella, the tibial tuberosity and the calcaneal insertions of the Achilles tendon and plantar fascia. In addition, bone oedema at the pubic symphysis and L5 spinous process was scored. The total enthesitis score was therefore 0–24.

c) Soft tissue inflammation: tenosynovitis was scored as present or absent as a single score for each hand and wrist and each foot and ankle, giving a tenosynovitis score out of 4. Bursitis was scored on each side as present or absent in the subdeltoid, greater trochanteric and retrocalcaneal bursae, giving a score out of 6.

2. Axial

The cervical, thoracic and lumbar spines were each scored for bone oedema using the sagittal T2-FS sequences. A single score of 1 or 0 denoting presence or absence was given for vertebral body oedema and for posterior element oedema in each region. The right and left sacroiliac joints were scored for presence or absence of subchondral oedema (0/1) and separately a score for presence or absence of erosion or sclerosis (0/1). Obvious Modic type I degenerative changes were not separated.

3. Image quality

Image quality was assessed with a subjective 1 to 4 scale as well as assessment of joint coverage and robustness of fat suppression. Image quality was scored as follows: 1, inadequate; 2, adequate; 3, good; and 4, very good.

### Clinical scoring

Clinical scoring of joint tenderness and swelling was performed by a single Rheumatology Early Arthritis Clinic Registrar for the same appendicular joints as for MRI and summed in the same way. Swollen and tender joint scores were not calculated for healthy volunteers. Axial joints were not scored for tenderness as published clinical enthesitis scoring tools do not typically include axial skeleton tenderness assessment.

### Statistical analysis

Statistical analysis was carried out with R version 3.6.1 [12] and using the packages dplyr [13] and ggplot2 [14].

Each anatomical area was analysed independently. Participants were divided into three groups (RA, non-RA and HC) based on their diagnosis at 1-year follow-up. Only consensus scores were used when comparing between different groups.

Synovitis scores in the joints were summed to give one overall score for each patient. Due to the small sample size in each group, non-parametric tests were used to compare scores. The Kruskal-Wallis test was used to compare the three different groups. If a *p*-value < 0.05 was obtained, pairwise Mann-Whitney *U*-tests with the Benjamini and Hochberg correction were used to determine which groups showed a statistically significant difference.

This process was repeated for erosion scores in joints, tenosynovitis scores in tendons, bursitis scores in the bursae and enthesitis scores in the entheses.

The proportion of patients in each group (RA, non-RA and HC) showing pathology in the vertebral body of each of the cervical spine, thoracic spine and lumbar spine was calculated. This was repeated for patients showing pathology in the posterior elements of each spine region. Similarly, the proportion of patients showing erosion in the SIJ joints and the proportion showing oedema in the SIJ were calculated.

The proportion of patients showing pathology in these areas was compared between groups (RA, non-RA and HC).

Receiver operating characteristic (ROC) curve analysis was performed on WBMRI joint synovitis and erosion scores, tender joint count (TJC) and swollen joint count (SJC), including calculating the area under the curve (AUC). ROC analysis was performed to identify the optimal threshold for diagnosis of rheumatoid arthritis from other forms of arthritis. The threshold value that maximised the Youden index was used as the optimal threshold for each method. ROC curves for MRI-based synovitis and erosion scores, TJC and SJC were compared to each other using the method of DeLong et al. [15] to determine which scoring method gave the best overall performance. Healthy controls were omitted from this analysis as clinical scores were not available.

Mean image quality scores were compared across each joint and region in the spine.

## Results

Fifty-five participants were included in this study comprising 18 patients with RA (10 cyclic citrullinated peptide (CCP) negative RA patients and 8 CCP positive RA patients), 28 patients with non-RA and 9 HC’s. The non-RA group consisted of the following subtypes: 10 persistent undifferentiated arthritis (pUA), 8 resolved undifferentiated arthritis (rUA), 5 spondyloarthritis (SpA), 4 undifferentiated arthritis and 1 crystal arthritis. Patients with undifferentiated disease did not fulfil published diagnostic criteria for known disease types, such as RA. A 10th healthy control was imaged but subsequently removed from the analysis, as they were diagnosed with both mechanical origin bilateral plantar fasciitis and a non-inflammatory spinal condition. Spine-only imaging was obtained for two patients in the non-RA group, meaning that two subjects’ data was omitted from analysis of the joints, bursae, tendons and entheses. A further three patients in the non-RA group could not be scored for tenosynovitis due to suboptimal positioning of the hands. The characteristics of patient and healthy controls are shown in Table 2.

**Table 2:** Clinical patient characteristics

	RA	Non-RA	HC
*N*	18	28	9
Female gender (%)	11 (61%)	15 (54%)	6 (67%)
Age	49.5 (44–55.5)	40.5 (28.5–48.5)	40 (32–50)
Clinical swollen joint score	2.5 (1–4)	0 (0–0)	NA
Clinical tender joint score	6.5 (3–13.3)	2 (0.5–6)	NA

Values shown are median (interquartile range (IQR))

### Appendicular joints

Median synovitis scores in each group were 6 (interquartile range (IQR) 1.5–9) for the RA group, 1.5 (IQR 0–3) for the non-RA group and 0 (IQR 0–1) for the HC group, giving statistically significant differences between the groups (*p* < 0.001). Pairwise testing showed differences in the number of joints affected between healthy controls and RA patients and between non-RA patients and RA patients (Table 3). No statistically significant difference was found between subjects in the HC group and the non-RA group. Erosion scores could distinguish between those in the RA group and the HC group, but not between either group and the non-RA group.

**Table 3:** Comparison of joint scores between different patient groups

	RA	Non-RA*	HC	*p*-value RA vs non-RA	*p*-value RA vs HC	*p*-value non-RA vs HC
Synovitis MRI scores	6 (1.5–9)	1.5 (0–3)	0 (0–1)	0.004	0.004	0.10
Erosion MRI scores	1 (0–3.75)	0 (0–1)	0 (0–0)	0.07	0.038	0.08
Swollen joint count	2.5 (1–4)	0 (0–0)	NA	< 0.001	N/A	N/A
Tender joint count	6.5 (3–13.3)	3 (0.5–6)	NA	0.13	N/A	N/A
Bursitis	2 (0–2.75)	1 (0–1)	0 (0–0)	0.043	0.028	0.043
Enthesitis	1.5 (0–3.75)	1 (0–2.75)	0 (0–0)	0.99	0.031	0.010
Tenosynovitis	1 (0–1)	0 (0–0.5)	0 (0–0)	0.88	0.09	0.35

Columns 2–4 show median (interquartile range) of each group. Columns 5–7 show *p*-value of pairwise Mann-Whitney *U*-test between each set of groups with correction for multiple comparisons. Asterisk symbol indicates that two patients in the non-RA group had spine-only imaging, and so these have been removed for this analysis

**Fig. 6:**
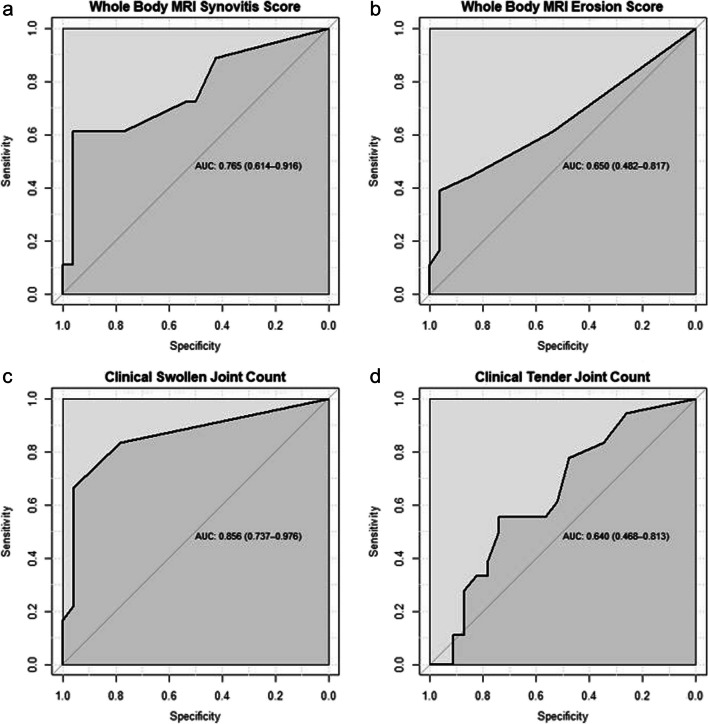
ROC curves for MRI and clinical scoring

AUCs of MRI synovitis and erosion scores, SJC and TJC were 0.77 (95% confidence intervals (CI), 0.61–0.91), 0.65 (95% CI, 0.48–0.82), 0.86 (95% CI, 0.74–0.98) and 0.64 (95% CI, 0.49–0.81), respectively. This showed that MRI synovitis scores had a higher AUC than TJC when used to differentiate RA and non-RA patients, but a lower AUC than SJC although there was no statistically significant difference between the ROC curves (*p* = 0.212). Table 4 and Fig. 6 give further results from the ROC analysis.

**Table 4:** AUC obtained from ROC of each scoring method. Optimal threshold is defined as that which maximises the Youden index

Scoring method	AUC (95% CI)	Optimal threshold (95% CI)	Sensitivity at threshold (95% CI)	Specificity at threshold (95% CI)
MRI whole-body synovitis score	0.765 (0.614–0.916)	5.5 (5.5–5.5)	61.1% (38.9–83.3%)	96.2% (88.5–100%)
MRI whole-body erosion score	0.650 (0.482–0.817)	1.5 (1.5–1.5)	44.4% (22.2–66.7%)	84.6% (69.2–96.2%)
Swollen joint count	0.856 (0.737–0.976)	1.5 (1.5–1.5)	66.7% (44.4–88.9%)	95.7% (87.0–100%)
Tender joint count	0.640 (0.486–0.813)	5.5 (5.5–5.5)	55.6% (33.3–77.8%)	73.9% (56.5–91.3%)

### Bursae, entheses and tendons

Non-parametric Kruskal-Wallis tests and subsequent pairwise Mann-Whitney *U*-tests showed significant differences between all three groups following scoring of bursitis (RA median = 2 IQR, 0–2.75, non-RA median = 1 IQR 0–1, HC median = 0, IQR 0–0, RA vs non-RA *p* = 0.038, RA vs HC *p* = 0.028, non-RA vs HC *p* = 0.043) (Table 4). Scoring of enthesitis from images showed statistically significant differences between the HC group and both patient groups but could not find any difference between the RA and the non-RA IA group (RA median = 1.5 IQR, 0–3.75, non-RA median = 1 IQR 0–2.75, HC median = 0 IQR 0–0, RA vs non-RA *p* = 0.99, RA vs HC *p* = 0.031, non-RA vs HC *p* = 0.010). No statistically significant differences were found between the three groups when looking at tenosynovitis.

### Axial joints

There was no significant difference in pathology in the vertebral bodies of the spine between either of the groups. Table 5 shows the amount of pathology seen in each area of the spine across each group. No pathology was seen in the posterior elements in any of the groups.

**Table 5:** Amount of pathology seen in vertebral bodies in each group expressed as an absolute value and percentage

Group	*N*	Cervical spine	Thoracic spine	Lumbar spine
RA	18	0 (0)	1 (5.6%)	0 (0%)
Non-RA	28	1 (3.6%)	2 (7.1%)	1 (3.6%)
Healthy control	9	1 (11%)	1 (11%)	0 (0%)

Scoring of the SIJs could also not distinguish between different groups, with those patients in the HC group proportionally having more pathology in the SIJ.

### Image quality

Image quality was high with all areas having a mean score of 3 (good) or higher. The lowest mean image quality was found in the wrist (3.22), whilst the highest was in the hip (3.86).

## Discussion

The importance of high-quality imaging with the use of appropriate sequences is understood by rheumatologists and radiologists alike, with recent guidance helping to establish the essential components of whole-body MR imaging [6, 16]. WBMRI can play a key role in the management of IA due to its sensitivity in in detecting synovitis, soft tissue inflammation and bone marrow oedema [17]. However, the study needs to be performed in the shortest feasible time, to ensure patient compliance and cost-effectiveness.

Development of MRI sequences has led to an increase in the differing methods of achieving fat suppressed images and the speed they can be achieved. The chemical shift water-fat separation proposed by Dixon [18] has become increasingly prevalent due to refinement of the technique to reduce B0 field inhomogeneity artefact [19] and increase the speed of acquisition [20]. The sequence can be of particular use in whole-body multi-joint imaging as it will obtain high-resolution post-gadolinium 3D images with robust fat/water suppression over a large field of view. One of the drawbacks of using the Dixon technique is an artefact known as water-fat swapping, but this can be mitigated by looking at both calculated images together. It does, however, remain difficult to interpret if the boundary occurs at the level of the joint, in which case the out-of-phase imaging is useful.

Standard whole-body imaging, where the patient is imaged head to toe, has been used in the assessment of rheumatological disease for some time [21] and particularly in inflammatory arthropathy. However, readability of the individual joints when using this whole-body technique has been raised as an issue previously; the literature states readability of around 70% [22] with the remainder of the imaging not considered of adequate quality to score, with the hands and feet often being the most affected by artefact. This is problematic because these peripheral joints will often be the first site to develop synovitis. The scan time is long compared with single joint imaging, and therefore, movement artefact often becomes a problem, particularly towards the end of the study.

The protocol we have developed allows robust high-resolution imaging of the peripheral joints, thanks to utilising a targeted “whole-body” approach. Several features allow the protocol to limit the scan time and maximise the image quality, providing benefits in terms of patient comfort and compliance, as well as accuracy for joint assessment. These include imaging of the hands, wrists, hips and pelvis in a single acquisition and utilising 3D near isotropic sequences to allow MPR and obviate the need for multiple plane acquisition. As well as using the post-gadolinium water-sensitive imaging for assessing synovitis, we used the in-phase and fat-sensitive sequences obtained from the 3D-Dixon acquisition to provide bone detail, for assessment of erosions. The utility of these sequences for erosion demonstration has recently been shown [23].

The total image acquisition time for whole body in our study was under 20 min (17 min 5 s) which is strongly in its favour given the amount of data obtained, including assessment of the axial skeleton and bone marrow assessment for osteitis. It is comparable, if not better, than an ultrasound scoring assessment which may average 30 min for hand and feet assessment and considerably longer if other areas were also included, as in the MRI protocol [24].

This protocol could be further optimised by the use novel MR acceleration technologies, including simultaneous multislice and AI-based reconstruction technologies such as Siemens’ Deep Resolve that have become available since this data was acquired. These technologies allow shorter image acquisition times with no loss of signal-to-noise ratio in the images. The saved time could be used to acquire images in other anatomical locations such as the elbows, or images with increased resolution.

Several other WBMRI protocols have been developed, although several of these require significantly longer scan times than the one we have developed here [22, 25, 26]. A protocol developed by Kamishima et al. could be acquired in a similar time frame (30 min) and also showed moderate to excellent image quality, however does not include any imaging of the spine, which we have managed to include in this protocol [11] .

Several findings from the study show its utility for further development and use in clinical practice. In patients presenting with IA, baseline WBMRI joint scores were significantly higher in those who were diagnosed at 12 months with RA suggesting that this technique may allow very early detection of those who will follow an RA phenotype. This protocol would be particularly useful in patients presenting with peripheral IA whilst concomitantly reporting possible inflammatory spinal symptoms, in whom it is important to rule this in/out in terms of diagnostic label and thus eligibility for biologic therapy .

Between groups, MRI images scored for synovitis identified differences in the number of joints affected between healthy controls and RA patients and between non-RA patients and RA patients. Scoring of bursitis and enthesitis was able to show differences between the HC group and both patient groups, although tenosynovitis was not useful.

Spine and SIJ imaging could not distinguish between different groups, with the HC group proportionally showing more abnormality in the SIJ. However, the presence of subchondral oedema and sclerosis at the SIJs is well recognised in healthy controls and represents a diagnostic problem for the classification of the axial spondyloarthropathies [27, 28].

In terms of image quality, over 95% of the target joints were visualised. The majority of joints not viewed were in the forefoot due to tall patients beyond the table movement limits. The lowest mean image quality was found in the wrist whilst the highest was in the hip, likely due to the hands being imaged in a large FOV.

One limitation of our study is the use of the T1 post-gadolinium imaging for the assessment of bone oedema at enthesis sites. Whilst T1-FS post-gadolinium imaging has been shown effective for the assessment of bone oedema compared with conventional water-sensitive sequences such as T2-FS [23, 29], studies using this technique have utilised SE sequences. The use of gradient echo imaging for detecting bone oedema is recognised as being less sensitive and should be evaluated in comparison to conventional sequences before relying on this protocol for the demonstration of bone oedema of enthesitis [23, 30]. This is one reason why we continued to employ T2-FS imaging for the assessment of bone oedema in the axial skeleton. Similarly, whilst our study has targeted the assessment of synovitis and erosions in the peripheral joints, we would be cautious in recommending the technique for the assessment of subchondral oedema at joints. However, without further modification, the protocol might be usefully used to include other joint features of arthritis such as osteophyte and subchondral sclerosis and cyst formation.

One area of future development for this protocol is the inclusion of a T2-FS 3D sequence in the hands/pelvis. This could likely be added without increasing scan time to over 30 min whilst achieving higher quality imaging of the hand.

Other limitations include the necessity for administration of intravenous contrast agent and the variable time after contrast injection between joints. The shoulders are scanned early in the study and so may demonstrate incomplete synovial enhancement whilst the feet are scanned late in the study and may demonstrate more florid synovial fluid enhancement. This can lead to false positives in the joints imaged last.

To keep the duration of the study as low as possible, a limited spine sequence was used which only covered laterally as far as the pedicles bilaterally. At the expense of additional time, T1 imaging of the spine could be incorporated into the protocol, which would be expected to provide additional information relating to structural changes of SpA. Additionally, the absence of the costovertebral joints in the spine acquisition is a key limitation, and this could also be added to the protocol at the cost of additional time.

Finally, this protocol does not include any imaging of the elbows. Again, this could be added at the cost of additional imaging time. These were not included in this protocol as to acquire good quality elbow imaging would also require re-positioning the patient, further adding to scan time.

In summary, this whole-body multi-joint MRI technique is feasible to use in a clinical setting and produces good quality images. It has the potential to differentiate between RA, non-RA and HC at early presentation. It is potentially useful in identifying disease “load”, including sub-clinical disease and extra-articular inflammation. The technique is transferrable to other MRI scanners and to other patient cohorts where assessment of global joint-based disease is desirable.
